# Characterization and Optimization of the Alkaline Hydrolysis of Polyacrylonitrile Membranes

**DOI:** 10.3390/polym11111843

**Published:** 2019-11-08

**Authors:** Leyre Pérez-Álvarez, Leire Ruiz-Rubio, Isabel Moreno, José Luis Vilas-Vilela

**Affiliations:** 1 Departamento de Química Física (Laboratorio de Química Macromolecular), Universidad del País Vasco (UPV/EHU), B Sarriena s/n, 48940 Leioa, Spain; joseluis.vilas@ehu.eus; 2 BCMaterials, Bizkaia Science and Technology Park, Building 500-1st Floor, 48160 Derio, Spain; 3 Macromolecular Chemistry Group (LQM), Organic Chemistry II Department, Faculty of Science and Technology, University of the Basque Country, 48940 Leioa, Spain; mariaisabel.moreno@ehu.eus

**Keywords:** polyacrylonitrile, surface modification, hydrolysis

## Abstract

There is currently an increasing interest in the development of polyacrylonitrile (PAN)-based membranes with new and enhanced properties which are of special importance in the processes of pervaporation, purification, and water treatment. Thus, the optimization of the functionalization of PAN membranes and its effect on their morphology, hydrophilicity, and mechanical properties plays an essential role in a wide range of applications. In this paper, the alkaline hydrolysis of asymmetric PAN membranes was investigated in order to get carboxyl-enriched surfaces that are of a great interest for more demanding subsequent modifications. The process was monitored using –C=NH intermediate bonds, which could be observed during the hydrolysis reaction by X-Ray photoelectron spectroscopy (XPS) and Fourier-transform infrared spectroscopy (FTIR) before the formation of carboxyl and amide groups. The amount of introduced carboxylic acid groups could be determined by thermogravimetric analysis (TGA) and by the interaction with toluidine blue O (TBO) dye. Hydrolysis was revealed as a simple way to modulate hydrophilicity (decreasing contact angle from 60 to 0° for reaction times from 0–3 h) and the mechanical properties of PAN membranes.

## 1. Introduction

Polyacrylonitrile (PAN) is an inexpensive and widely-used polymer for several applications such as foams [1], nano-sensors [2], biomaterials [3], ultrafiltration [4], and pervaporation [5]. Specifically, PAN has been successfully used as one of the most important materials for membrane preparation due to its good thermal and mechanical stability, high abrasion resistance, and its facility for being prepared as highly porous polymer [6]. In this sense, PAN is traditionally employed as a semipermeable membrane for hemodialysis due to its good properties [6]. However, its hydrophobic nature limits its biocompatibility and consequently its applicability as a biomaterial [7]. Fortunately, PAN is a relatively active polymer, which makes it easy to be modified. The most employed modification methods of PAN membranes include plasma treatment [8] and hydrolysis [9]. Alkaline hydrolysis with strong basic solutions in which –CN groups on the PAN membrane surface turn into –COO− groups [10] has been widely used for PAN modification due to the promoted uniform distribution of functional groups along the chain. Thus, alkaline hydrolysis of PAN is used on a large scale to produce water-soluble additives and superabsorbents [11] for engineering and consumer products [12]. PAN hydrolysis has also been employed in the preparation of highly hydrophilic nanofiltration membranes with controlled antifouling and rejection properties [13]. More recently, PAN hydrolysis has been employed as a starting modification process for the preparation of more complex multifunctional membranes such as biofunctionalized membranes [14], composites [15], and polyelectrolyte multilayers [16] with diverse purposes.

However, the properties of hydrolyzed PAN are strongly determined by the extent of the hydrolysis reaction [17]. Consequently, although the reaction has been studied for decades [10,17,18,19], it continues to be a significant field of study for practical applications; deeper insight into the relation between extent of hydrolysis and the macroscopic properties is still required. Based on this, this work presents a study of the effect of the surface treatment of PAN membranes by alkaline hydrolysis on their hydrophilicity and mechanical properties, which have been poorly studied in previously reported works.

## 2. Materials and Methods

### 2.1. Materials

Polyacrylonitrile (PAN) of average molecular weight *M*_w_ ≈ 150,000 g mol^−1^ (Sigma-Aldrich, St. Louis, MO, USA), *N*,*N*–dimethylformamide (DMF, 99.8%, Sigma-Aldrich, Darmstadt, Germany), and lithium chloride (LiCl, 99%, Sigma-Aldrich, Darmstadt, Germany) were used as polymer, solvent, and pore former, respectively, for the fabrication of membranes. Sodium hydroxide (pellets, Panreac, Barcelona, Spain) was used in the hydrolysis of PAN membranes. Toluidine blue O (TBO) dye was purchased from Sigma-Aldrich (St. Louis, MO, USA). All reactants were used without further purification.

### 2.2. PAN Membrane Preparation

PAN solution was prepared by mixing 14 wt % of PAN and 2 wt % of LiCl additive in DMF at 60 °C. The solution was allowed to stand while stirring overnight, then it was cast on a microscope cover glass of 22 mm thickness by spin-coating (speed (rpm): +2000; sample volume (μL): 0.2). The cast film was gelled in a coagulant bath containing water and was later washed with milliQ water. Hydrolysis of PAN membranes was carried out in a 1.5 M NaOH solution in a thermostatic bath at 45 °C. PAN membranes were collected and rinsed with deionized water until reaching a neutral pH. The porosity (*ε*) of prepared membranes was determined by weighing according to Equation (1) [20]:

(1) $$\varepsilon =\frac{\frac{m_{wet}-m_{dry}}{\rho_{w}}}{\frac{m_{wet}-m_{dry}}{\rho_{w}}+\frac{m_{dry}}{\rho_{p}}}\times 100\%$$

where *ρ_w_* and *ρ_p_* are the density of the wetting solvent (distilled water) and the polymer (at 20 °C), and *m_wet_* and *m_dry_* are the wet mass and the dry mass of membranes.

### 2.3. Characterization Techniques

#### 2.3.1. Fourier-Transform Infrared Spectroscopy (FTIR)

The molecular structure of PAN membranes was analyzed by Fourier-transform infrared spectroscopy (FTIR). FTIR spectra were acquired using a NEXUS 670 spectrophotometer (Nicolet Thermo Instruments Inc., Waltham, MA, USA). Dried samples were scanned in an attenuated total reflectance (ATR) mode at frequencies from 400 to 4000 cm^−1^ and with 32 scan times per spectrum. The nominal resolution was set to 4 cm^−1^.

#### 2.3.2. UV–Vis Spectroscopy

The hydrolyzed ratio of the PAN membranes was analyzed through the increase of carboxyl group concentration. These carboxylic groups, formed during the hydrolysis, react with TBO through the formation of ionic complexes. The hydrolyzed membranes were immersed in a 0.5 mM TBO aqueous solution (pH = 10) for 12 h at room temperature in order to allow complex formation. Then, PAN membranes were cleaned with a 0.1 mM NaOH solution to remove the excess of TBO. Finally, the TBO bonded to the membranes was desorbed by immersion of the substrates in a 4 mL 50% acetic acid solution for 10 min. The absorbance at 633 nm was recorded by a UV–Vis spectrophotometer (UV-2450, Shimadzu, Kioto, Japan). The amount of the carboxyl groups was calculated by using a calibration curve of TBO/50% acetic acid solution recorded in the same conditions (A = 75301.9 M (mol L^−1^) + 877.8, R^2^ = 0.9993). A complexation ratio of 1:1 mol of TBO/carboxylic acid was considered for the calculation [21].

#### 2.3.3. X-Ray Photoelectron Spectroscopy (XPS)

XPS measurements were performed in a SPECS system (SPECS Surface Nano Analysis, Berlin, Germany) equipped with a Phoibos 150 1D-DLD analyzer (SPECS, Berlin, Germany) using a monochromatic Focus 500 X-ray source with an Al/Ag dual anode.

#### 2.3.4. Contact Angle

The contact angle of the membranes was measured using the optical system Dataphysics OCA 15EC (Dataphysics, Filderstadt, Germany). Milli-Q water was dropped on each sample (2 μL/drop). Reported data are the average of 10 measurements.

#### 2.3.5. Scanning Electron Microscopy (SEM)

The surface and thickness of the membranes were analyzed by scanning electron microscopy with a HITACHI S-4800 microscope (150 s, 20 mA, 15 kV) (HITACHI, Krefeld, Germany). The cross-sectional images of the films were obtained after fracturing the cooled films in liquid N_2_ and were uniformly overlaid with gold.

#### 2.3.6. Mechanical Properties

The study of the mechanical properties of 2 cm × 5 cm sized wet membranes was performed in an AGS-X Universal Testing Machine from Shimadzu (Kioto, Japan) at a constant jack speed of 5 mm s^−1^.

#### 2.3.7. Thermogravimetric Analyses (TGA)

Thermal stability was studied with a Thermal Gravimetric Analyzer (TGA) TGA/SDTA 851e Metter Toledo apparatus (Gießen, Germany) from 25 to 700 °C at a heating rate of 10 °C/min while under nitrogen flow (20 mL/min).

## 3. Results

### 3.1. Modification of Surface Composition

**Figure 1:**
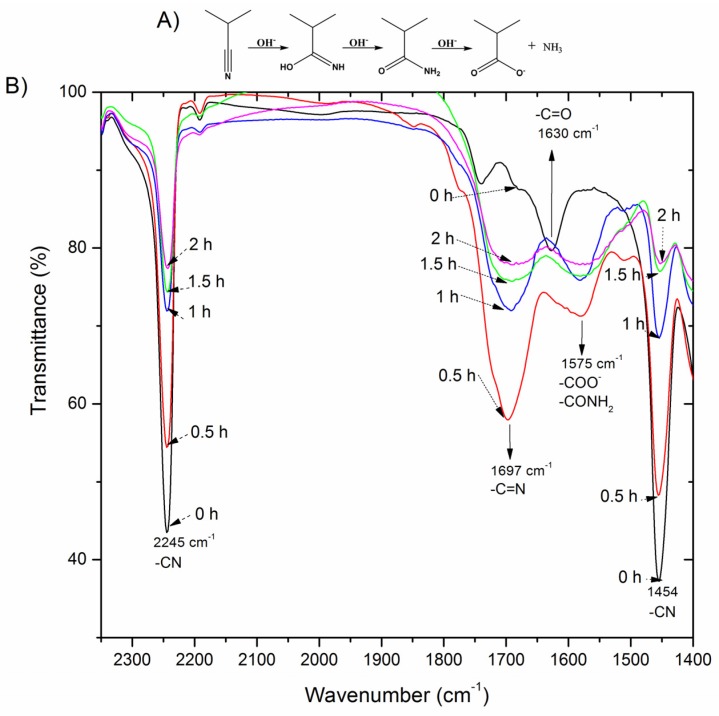
(**A**) Simple schematic representation of the mechanism of PAN (Polyacrylonitrile) hydrolysis, and (**B**) FTIR (Fourier-transform infrared spectroscopy) spectra of PAN membranes for different hydrolysis times.

PAN surface modification was carried out using a hydrolysis reaction through addition of NaOH according to the conditions described above. It is well known that the mechanism of the hydrolysis reaction of PAN consists of two different stages [22]. In the first stage, the attack of the hydroxyls on nitrile groups takes place, generating an amide moiety. In the second step, the addition of another hydroxyl group on the amide causes the disappearance of –CONH_2_ groups and the formation of the carboxylic acids. However, this mechanism has been extensively discussed and various investigations have revealed the existence of intermediate stages [10,23]. Thus, when the reaction starts (Figure 1), the –C≡N triple bond becomes a double bond (–C=N) before the formation of the amide groups; however, experimental evidence of this intermediate bond has rarely been reported [10]. The infrared spectra of hydrolyzed PAN membranes were obtained in order to study and confirm the hydrolysis reaction mechanism. Apart from the characteristic peak at 1630 cm^−1^ of the carbonyl group (C=O), untreated PAN membranes exhibit two characteristic peaks at 1454 and 2245 cm^−1^, corresponding to the C–N stretching of the –C≡N group (Figure 1) [9,11]. As hydrolysis takes place, these bands clearly decrease [9,16] while the appearance of two new peaks at 1697 and 1575 cm^−1^ is observed. These new bands, which have only been reported by a few researchers [24], correspond to the intermediate –C=N and amide (–CONH_2_) moieties, respectively, and their intensities vary according to the reaction mechanism proposed by Litmanovich et al. [10]. Thus, –C=N stretching (1697 cm^−1^) increases for short modification times (0.5 h) corresponding to the first stage of hydrolysis, but rapidly decreases for higher reaction times in the second stage as amide and carboxylate groups are formed, verifying the above described mechanism.

**Figure 2:**
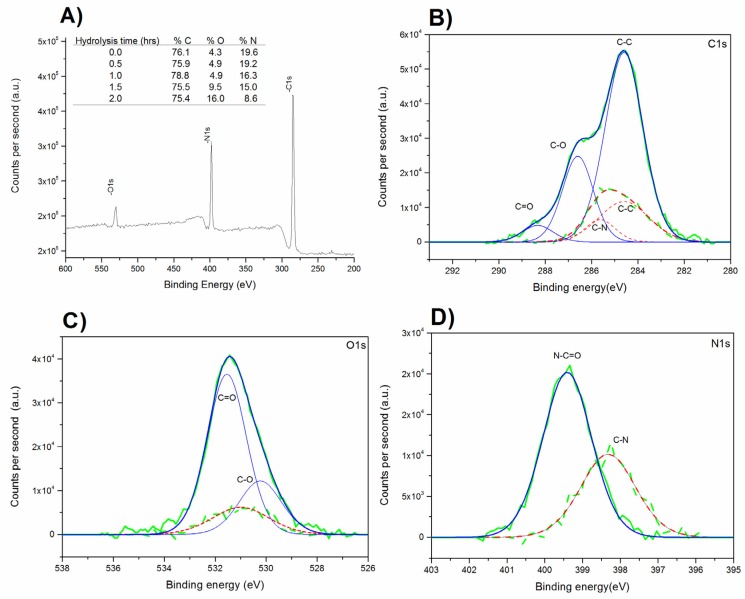
(**A**) XPS (X-ray photoelectron spectroscopy) curves of PAN membranes and atomic surface composition of PAN membranes at different hydrolysis times. Deconvolution of untreated (--, **red**) and hydrolyzed (—, **blue**) (1.5 h) PAN membranes for (**B**) C1s, (**C**) O1s, and (**D**) N1s peak.

The study of the elemental composition of the surface of PAN membranes at different hydrolysis times was performed by XPS. According to the analysis of the elemental composition shown in Figure 2A, an overall increase in oxygen content and a decrease in nitrogen content with increasing treatment time were observed, especially for long hydrolysis times. This trend is consistent with the proposed mechanism of the hydrolysis reaction, confirming that N_1s_ content and O_1s_ content remain almost constant in the first stage of the reaction while they decrease and increase, respectively, in the second stage due to the loss of –C=N and –CONH_2_ moieties and the formation of –COOH groups.

To confirm the appearance of the intermediate amide bonds, the formation of carboxyl groups, and that the decreasing of nitrogen corresponds to the loss of cyano groups, high resolution C1s, O1s, and N1s peaks were also analyzed (Figure 2B–D, respectively) according to the mechanism described above.

Deconvolution of C1s (Figure 2B) shows a peak at 285.6 eV corresponding to the C–N bond which is not present in hydrolyzed PAN. However, hydrolyzed samples display two new peaks at 286.5eV and 288.5 eV ascribed to the formed C–O and C=O bonds respectively [22,25]. In Figure 2C, the deconvolution of O_1s_ is displayed only in the case of hydrolyzed samples; two peaks corresponding to the new C=O and C–O bonds were formed in the reaction at 531 eV and 530 eV, respectively. In addition, N_1s_ deconvolution (Figure 2D) clearly shows the disappearance of the peak attributed to C≡N at 398.3 eV and the formation of a new peak of the C=N bond at 399.5 eV, confirming the formation of intermediate C=N bonds and thus the mechanism described above.

The TBO dye colorimetric method is a widely-used method for the quantification of surface carboxyl groups. This cationic compound interacts electrostatically with negatively charged groups, which can be easily detected by light absorption in the range of the visible blue wavelength. Although TBO technique is known as one of the most straightforward methods for surface charge quantification, the results obtained showed a high standard deviation that could be ascribed to some non-specific adsorption of dye to the membrane surface; this has been also observed in several polymeric membranes [26].

**Figure 3:**
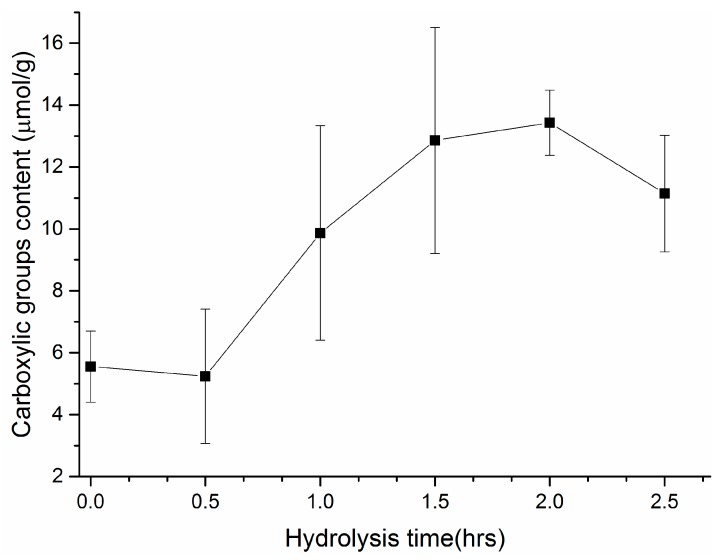
Content of carboxylic groups in hydrolyzed PAN membranes for different treatment times determined by Toluidine blue O (TBO) colorimetric method.

An increase in carboxyl group amount with hydrolysis reaction time was observed, but it remained almost constant during the initial stage and when the hydrolysis time was longer than 1.5 h, accordingly with the evolution of O content determined by XPS during the reaction. Thus, a hydrolysis time of 1.5–2 h could be considered as the optimal treatment time, with a maximum amount of carboxyl groups of approximately 7 μmol/g in the samples (Figure 3).

### 3.2. Properties of Modified Membranes

**Figure 4:**
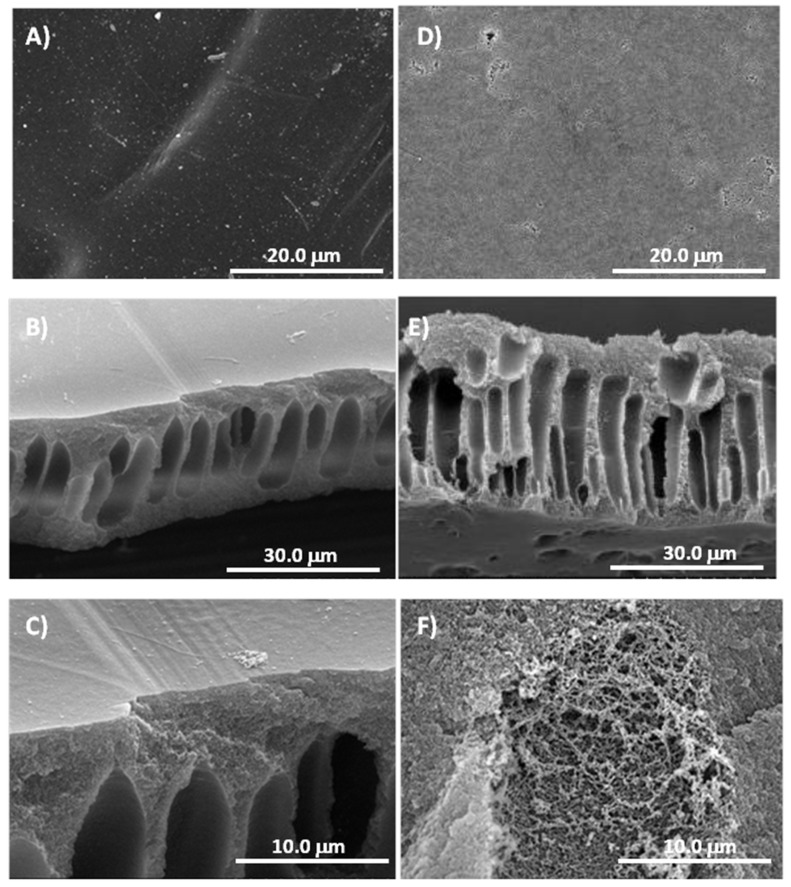
SEM images of PAN membrane (**A**) untreated surface × 2.5 k, (**B**) untreated cross-section × 1.5 k, (**C**) amplified untreated cross-section × 5.0 k and hydrolyzed during 1.5 h × 1.5 k, (**D**) surface × 2.5 k, (**E**) cross-section × 1.5 k, and (**F**) amplified hydrolyzed cross-section × 4.5 k.

The morphology of PAN membranes was studied using SEM; images of their surfaces and cross-sections are shown in Figure 4. PAN membranes display the morphological characteristics of asymmetric membranes prepared by the phase inversion technique [4]. Because of the addition of LiCl, asymmetric cross-sections possess abundant amounts of macrovoids combining with macrovoid-free sections with finely formed porous (sponge-like) structures [4]. As can be seen in Figure 4, the surface hydrolysis reaction does not significantly modify the morphology of the membranes for reaction times lower than 1.5 h. In the same way, no changes in membrane porosity (ε) were measured when untreated (ε_mean_ = 83.2 ± 2.1%) and 1.5 h hydrolyzed membranes (ε_mean_ = 81.8 ± 1.5%) were compared.

**Figure 5:**
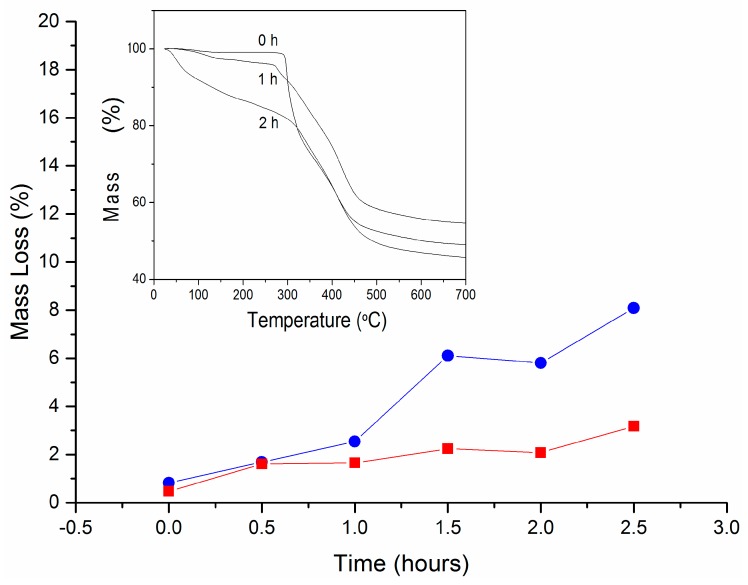
(•) Water and (■) –COOH mass loss (*w*/*w* %) of PAN membranes as a function of hydrolysis time, and thermogravimetric curves of hydrolyzed PAN membranes for the more representative hydrolysis times.

The modification of the material’s surface accelerates the thermal degradation of the membranes, as can be observed in Figure 5. All samples showed a main loss of weight between 290 and 460 °C, derived from the decomposition of the polymer into volatile products. However, two previous weight loss stages were differentiated before, at 25–120 °C and 200–300 °C. The first one, ascribed to water evaporation, allowed the determination of the water content of the prepared membranes (Figure 5). A significant weight loss between 200 and 300 °C was observed for hydrolyzed PAN membranes for 1 h or more. It is well known that carboxylic acid groups thermally decompose in this temperature range (–COOH → CO_2_) [27], which confirms the successful hydrolysis reaction of PAN membranes. The weight loss in this temperature range could easily be used as a rapid estimation of the number of carboxylic groups formed as a consequence of the hydrolysis treatment (Figure 5) [27].

**Figure 6:**
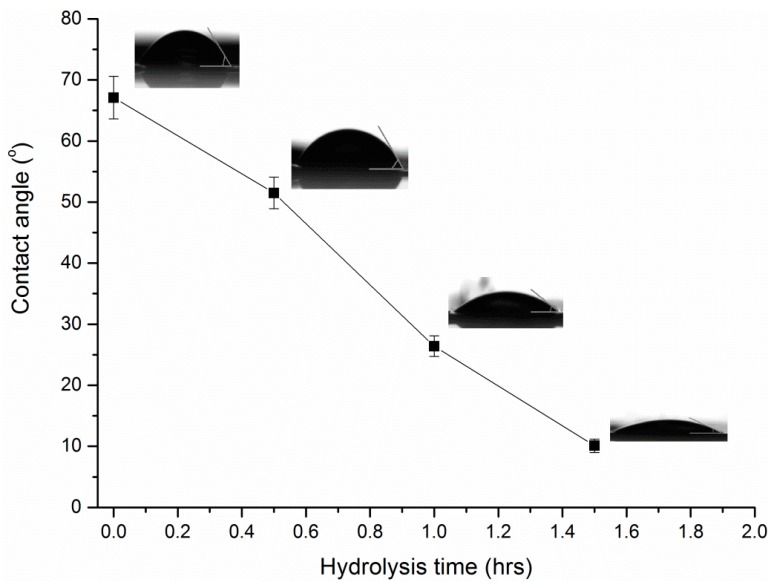
Contact angle of PAN membranes for different hydrolysis reaction times.

The hydrophilicity of the hydrolyzed surfaces of PAN was studied using the measurement of the contact angle, as is shown in Figure 6. The great reduction of the contact angle during hydrolysis, from 69.59 ± 4.99° for the untreated film to 10.1 ± 1.1° for the film hydrolyzed during 1.5 h, evidences the drastic modification of the material, leading to a highly hydrophilic surface. This variation, observed by several authors [28], is attributed to the introduction of hydrophilic groups such as carboxyl groups on the surface of the polymeric substrate.

**Figure 7:**
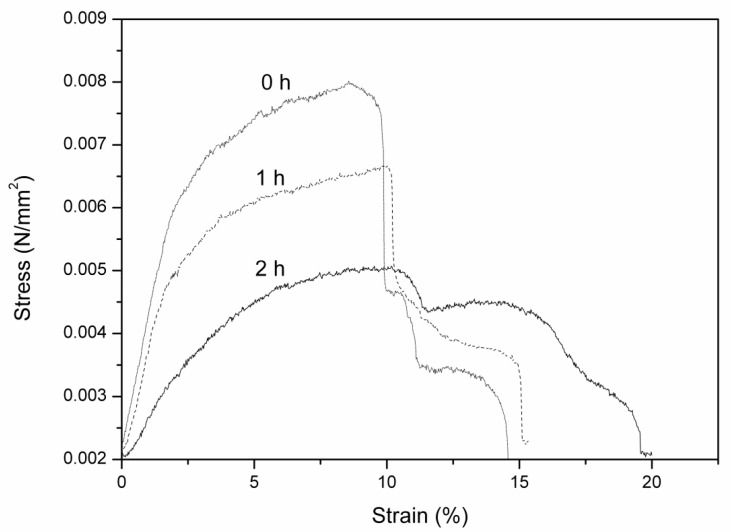
Assays of stress–strain of hydrolyzed PAN membranes.

The study of the mechanical properties of hydrolyzed PAN membranes as a function of the hydrolysis time was performed. The stress–strain curves obtained for each material are shown in Figure 7. It is noted that the Young module decreases as the hydrolysis time increases, indicating that the membranes become more deformable due to the higher water content [29] as a consequence of their higher hydrophilicity, as it is observed in contact angle and thermogravimetric studies. After hydrolysis reaction times higher than two hours, membranes displayed extremely poor mechanical properties and stress–strain curves could not be obtained.

## 4. Conclusions

Hydrolysis with NaOH is a simple and appropriate way of incorporating –COOH moieties to asymmetric PAN membranes. FTIR and XPS analyses made it possible to discriminate intermediate –C=N bond formation before –COOH and –CONH_2_ appearance during the modification of polyacrylonitrile membrane surfaces, and to monitor the progress of the reaction. The alkaline hydrolysis reaction did not alter the morphology and the porosity of the membranes for reaction times less than 1.5 h. Highly hydrophilic surfaces were obtained after hydrolysis treatment, displaying contact angles close to 0°. Toluidine blue adsorption could be used to estimate the concentration of the incorporated –COOH group in the membranes; however, non-specific adsorption of dye to the membrane surface was observed. Surface hydrolysis leads to a decrease in the Young module and an increase in the deformability of PAN membranes. The optimization of the hydrolysis reaction time allows the modulation of the morphological changes, surface functionalization with –COOH groups, hydrophilicity, and mechanical properties of PAN membranes.

## References

[1] Aubert J.H., Sylwester A.P. (1991). Morphological characterization of microcellular carbon foams. J. Mater. Sci..

[2] Jain S., Chattopadhyay S., Jackeray R., Singh H. (2009). Surface modification of polyacrylonitrile fiber for immobilization of antibodies and detection of analyte. Anal. Chim. Acta.

[3] Ren X., Akdag A., Zhu C., Kou L., Worley S.D., Huang T.S. (2009). Electrospun polyacrylonitrile nanofibrous biomaterials. J. Biomed. Mater. Res. Part A.

[4] Phadke M.A., Kulkarni S.S., Karode S.K., Musale D.A. (2005). Poly(acrylonitrile) ultrafiltration membranes. II. membrane morphology and permeation characteristics. J. Polym. Sci. Part B.

[5] Huang Y.H., Huang S.H., Chao W.C., Li C.L., Hsieh Y.Y., Hung W.S., Liaw D.J., Hu C.C., Lee K.R., Lai J.Y. (2014). A study on the characteristics and pervaporation performance of polyamide thin-film composite membranes with modified polyacrylonitrile as substrate for bioethanol dehydration. Polym. Int..

[6] Wang Z.G., Wan L.S., Xu Z.K. (2007). Surface engineerings of polyacrylonitrile-based asymmetric membranes towards biomedical applications: An overview. J. Membr. Sci..

[7] Yang M.C., Lin W.C.J. (2002). Surface modification and blood compatibility of polyacrylonitrile membrane with immobilized chitosan-heparin conjugate. Polym. Res..

[8] Tran T.D., Mori S., Suzuki M. (2007). Plasma modification of polyacrylonitrile ultrafiltration membrane. Thin Solid Films.

[9] Zhang G., Meng H., Ji S. (2009). Hydrolysis differences of polyacrylonitrile support membrane and its influences on polyacrylonitrile-based membrane performance. Desalination.

[10] Litmanovich A.D., Plate N.A. (2000). Alkaline hydrolysis of polyacrylonitrile. On the reaction mechanism. Macromol. Chem. Phys..

[11] Gupta M.L., Gupta B., Oppermann W., Hardtmann G. (2004). Surface modification of polyacrylonitrile staple fibers via alkaline hydrolysis for superabsorbent applications. J. Appl. Polym. Sci..

[12] Michels G., Sackmann G., Struss K. (2003). Process for the Preparation of Superabsorbent Polymers from Polyacrylonitrile Precipitation Polymers. U.S. Patent.

[13] Cornelissen E.R., Van den Boomgaard T., Strathmann H. (1998). Physicochemical aspects of polymer selection for ultrafiltration and microfiltration membranes. Colloids Surf. A.

[14] Liu T.Y., Lin W.C., Huang L.Y., Chen S.Y., Yang M.C. (2005). Hemocompatibility and anaphylatoxin formation of protein-immobilizing polyacrylonitrile hemodialysis membrane. Biomaterials.

[15] Gao C., Zhang M., Ding J., Pan F., Jiang Z., Li Y., Zhao J. (2014). Pervaporation dehydration of ethanol by hyaluronic acid/sodium alginate two-active-layer composite membranes. Carbohydr. Polym..

[16] Zhang G., Yan H., Ji S., Liu Z. (2007). Self-assembly of polyelectrolyte multilayer pervaporation membranes by a dynamic layer-by-layer technique on a hydrolyzed polyacrylonitrile ultrafiltration membrane. J. Membr. Sci..

[17] Dyatlov V.A., Grebeneva T.A., Rustamov I.R., Koledenkov A.A., Kolotilova N.V., Kireev V.V., Prudskov B.M. (2012). Hydrolysis of polyacrylonitrile in aqueous solution of sodium carbonate. Polym. Sci. Ser. B.

[18] Sanli O. (1990). Homogeneous hydrolysis of polyacrylonitrile by potassium hydroxide. Eur. Polym. J..

[19] Krentsel L.B., Kudryavtsev Y.V., Rebrov A.I., Litmanovich A.D., Platé N.A. (2001). Acidic Hydrolysis of Polyacrylonitrile: Effect of Neighboring Group. Macromolecules.

[20] Wei J., Qiu C., Tang C.Y., Wang R., Fane A.G. (2011). Synthesis and characterization of flat-sheet thin film composite forward osmosis membranes. J. Memb. Sci..

[21] Liu Y., He T., Gao C. (2005). Surface modification of poly(ethylene terephthalate) via hydrolysis and layer-by-layer assembly of chitosan and chondroitin sulfate to construct cytocompatible layer for human endothelial cells. Colloids Surf. B.

[22] Xu B., Wang X., Lu Y. (2006). Surface modification of polyacrylonitrile-based carbon fiber and its interaction with imide. Appl. Surf. Sci..

[23] Bao W., Xu Z., Yang H. (2009). Electrokinetic and permeation characterization of hydrolyzed polyacrylonitrile (PAN) hollow fiber ultrafiltration membrane. Sci. China Ser. B Chem..

[24] Deng S., Bai R., Chen J.P. (2003). Behaviors and mechanisms of copper adsorption on hydrolyzed polyacrylonitrile fibers. J. Colloid Interface Sci..

[25] Pirlot B.C., Willems I., Fonseca A., Nagy J.B., Delhalle J. (2002). Preparation and Characterization of Carbon Nanotube/Polyacrylonitrile Composites. J. Adv. Eng. Mater..

[26] Tiraferri A., Elimelech M. (2012). Direct quantification of negatively charged functional groups on membrane surfaces. J. Membr. Sci..

[27] Rosmaninho M.G., Jardim E., Moura F.C.C., Ferreira G.L., Thom V., Yoshida M.I., Araujo M.H., Lago R.M. (2006). Surface hydrolysis of postconsumer polyethylene terephthalate to produce adsorbents for cationic contaminants. J. Appl. Polym. Sci..

[28] Saren Q., Qiu C.Q., Tang C.Y. (2011). Synthesis and characterization of novel forward osmosis membranes based on layer-by-layer assembly. Environ. Sci. Technol..

[29] Mirbaha H., Arbab S., Zeinolebadi A., Nourpanah P. (2013). An investigation on actuation behavior of polyacrylonitrile gel fibers as a function of microstructure and stabilization temperature. Smart Mater. Struct..

